# Acute Appendicitis in a Man Undergoing Therapy for Mantle Cell Lymphoma

**DOI:** 10.1155/2012/868151

**Published:** 2012-03-29

**Authors:** Michael Linden, Ajay Gopal, Kerstin Edlefsen

**Affiliations:** ^1^Department of Laboratory Medicine and Pathology, University of Minnesota, MMC 806, Minneapolis, MN 55455, USA; ^2^Department of Medicine, University of Washington, Seattle, WA 98195, USA; ^3^Seattle Cancer Care Alliance, Seattle, WA 98109, USA; ^4^Department of Laboratory Medicine, University of Washington, Seattle, WA 98195, USA

## Abstract

A 71-year-old man was diagnosed with an aggressive mantle cell lymphoma and was started on six cycles of R-CHOP chemotherapy. Approximately two weeks after starting his first cycle of chemotherapy, he complained of severe right lower quadrant abdominal pain, and an abdominal CT scan demonstrated an enlarged appendix with evidence of contained perforation. The man underwent open appendectomy for acute appendicitis and recovered. The appendectomy specimen was submitted for routine pathological analysis. There was histologic evidence of perforation in association with an inflammatory infiltrate with fibrin adhered to the serosal surface; scattered small lymphoid aggregates were present on the mucosal surface. Although the lymphoid aggregates in the submucosa and lamina propria were rather unremarkable by routine histologic examination, immunohistochemistry revealed the lymphocytes to be predominantly Cyclin D1-overexpressing B cells. To our knowledge, this is the first reported case of acute appendicitis in association with appendiceal involvement by mantle cell lymphoma.

## 1. Introduction

 An appendectomy specimen collected in a clinical scenario supportive of acute appendicitis is a common sample received in gross rooms for routine pathology analysis. Indeed, the relative simplicity of the specimen makes it a rite of passage for medical students and new residents first approaching the pathology gross examination. While the pathologic examination of the appendix typically supports the clinical impression of acute appendicitis, routine histopathological analysis plays a crucial role in evaluating for occult pathologic conditions, including neoplasia. Carcinomas are the most frequent neoplasms encountered in the appendix; however, other less frequently encountered neoplasms include carcinoid tumors and lymphomas [1]. Lymphoma involving the appendix in association with acute appendicitis has been rarely described previously, with the majority of cases being Burkitt or large B-cell lymphoma [2–15]. Here we report an unusual example of acute appendicitis presenting in a patient undergoing treatment for mantle cell lymphoma.

## 2. Case Report

A 71-year-old man complained of a two week history of right-sided abdominal pain that started after he began his first round of chemotherapy with R-CHOP with pegfilgrastim for mantle cell lymphoma. He presented to the emergency department (ED) after he developed severe (9 out of 10) right lower quadrant abdominal pain, approximately 17 days after starting chemotherapy. A physical examination in the ED revealed tenderness most pronounced in the right lower quadrant, with guarding and rebound pain. Lymphadenopathy was also noted in the cervical and supraclavicular regions. Imaging by CT scan of the abdomen/pelvis revealed an enlarged appendix with disruption of the appendiceal wall and small foci of gas and fluid consistent with a contained perforation. No associated mass or tumor was observed in the imaging studies. Peripheral blood was collected and sent for complete blood count, which demonstrated a normocytic anemia with a relative neutrophilia.

The patient's mantle cell lymphoma had been recently diagnosed on the basis of involvement of bone marrow and cervical lymph nodes. Imaging studies revealed widespread lymphadenopathy and splenomegaly. The mantle cell lymphoma expressed CD5 and kappa light chain restriction by flow cytometry and expressed Cyclin D1 by immunohistochemistry. The mantle cell lymphoma was noted to have a high Ki-67-defined proliferative rate of 60–80%, a finding that suggested an aggressive clinical behavior. There was no prior documented involvement of the gastrointestinal system. Based on these findings, the patient was diagnosed with an aggressive mantle cell lymphoma, stage IVA. The treatment plan for the lymphoma included 6 cycles of R-CHOP (rituximab, cyclophosphamide, doxorubicin, vincristine, and prednisone) chemotherapy with pegfilgrastim support for induction, which was to be followed by high-dose consolidation and stem cell collection (mobilized, peripherally collected) in anticipation of potential future autologous bone marrow transplantation.

Several days before the patient was to receive the second cycle of R-CHOP; however, he presented with acute appendicitis and was referred urgently to general surgery. He was taken to the operating room on the same day for laparoscopic appendectomy, which was converted to open appendectomy with washout when it was directly observed that the appendix had perforated and had dense adhesions. Two small abscess cavities were identified, each of which drained purulent material on opening. His recovery from surgery was generally unremarkable other than mildly prolonged postoperative ileus and the presence of two small persistent abscesses by imaging postoperatively. He was discharged 9 days after his surgery on antibiotics and was continued on rituximab alone until he was able to return to his second cycle of CHOP roughly 1 month after his presentation with acute appendicitis.

**Figure 1:**
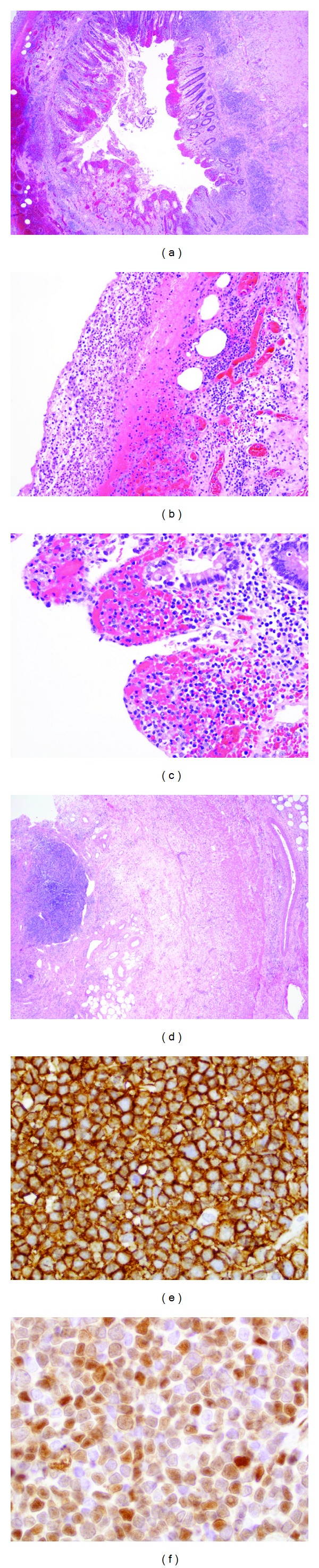
(a) Cross-section of appendix with disrupted mucosal surface and small lymphoid aggregates (hematoxylin and eosin, 2x objectives); (b) serosal surface of appendix with fibrinopurulent adhesions and dilated vessels (hematoxylin and eosin, 10x objective); (c) Inflamed mucosal surface of appendix (hematoxylin and eosin, 20x objective); (d) dense, thickened appendiceal and a lymphoid aggregate (also depicted in (e) and (f)) (hematoxylin and eosin, 2x objective); (e) CD20 immunostain, 50x oil objective; (f) cyclin D1 immunostain, 50x oil objective. All images were captured with an Olympus DP70 camera attached to an Olympus BX45 with a 1x U adapter.

The gross examination of the appendectomy specimen showed an enlarged appendix with a clear area of perforation and fibrous adhesions on the serosal surface. Representative sections were submitted for conventional histologic processing. While some portions of the appendiceal lumen had normal histologic findings, the most inflamed portions had a disrupted mucosal surface (Figure 1(a)). Scattered on the mucosal surface were small aggregates composed predominantly of small lymphocytes and few plasma cells, along with focal neutrophil-rich areas consistent with suppurative inflammation (Figures 1(a) and 1(c)). The appendiceal wall was thickened and fibrous (Figures 1(a) and 1(d)), and the inflamed serosa contained dilated vessels with a surface coated by fibrinopurulent debris (Figure 1(b)). Overall, the submucosal lymphoid aggregates appeared small (Figure 1(a)). They did not have discrete germinal centers and were composed predominantly of small- to intermediate-sized lymphocytes with high nuclear: cytoplasmic ratios, but without significant nuclear atypia. As the patient had a history of mantle cell lymphoma, we performed immunohistochemical studies on the lymphoid aggregates (Figure 1(d)). The lymphoid aggregates contained predominantly CD20-positive B cells (Figure 1(e)) in association with CD3-positive T cells, which were interspersed and predominantly at one edge (not shown). The neoplastic B cells expressed nuclear *cyclin D1* gene product (Figure 1(f)), but lacked expression of BCL-6 (not shown). The morphologic and immunophenotypic findings confirm that the patient's appendiceal specimen was involved by mantle cell lymphoma.

Unfortunately, while the patient recovered postoperatively, in the interim he developed circulating neoplastic lymphocytes in the peripheral blood. His mantle cell lymphoma proved to be refractory to multiple chemotherapeutic regimens. He became increasingly ill and, in discussion with his family, care was ultimately withdrawn. The patient died from his lymphoma approximately 6 months after his presentation with acute appendicitis.

## 3. Discussion

Mantle cell lymphoma is an intermediate-grade B-cell neoplasm composed of monomorphic small-to medium-sized lymphocytes with irregular nuclear contours and is characterized by overexpression of *cyclin D1* as a result of a t(11;14) (q13;q32) translocation. [16, 17] Most patients present with lymphadenopathy, often with spleen and bone marrow involvement. [16] Additionally, mantle cell lymphoma frequently involves the gastrointestinal tract, sometimes presenting as lymphomatous polyposis [18]. In many cases the involvement of the gastrointestinal tract is occult, requiring endoscopy for diagnosis.

 Lymphoma involving an appendectomy specimen has been previously described, albeit rarely [2–15]. We found less than 20 cases of acute appendicitis in association with lymphomatous infiltration in the English literature. A majority of cases represent Burkitt lymphoma, [3, 6, 8, 11, 15] with the remainder of cases representing large cell lymphoma [5, 9, 12] or without classification [2, 10, 13, 14]. To the best of our knowledge, this is the first case report of appendicitis in an appendix involved by mantle cell lymphoma. This is somewhat surprising, given the known tendency of mantle cell lymphoma to involve the gastrointestinal tract. It is noted that there is a single case report that describes finding in situ mantle zone lymphoma (i.e., scattered Cyclin D1 positive cells in the mantle zone) at the time of retrospective review of an appendectomy specimen from a patient subsequently diagnosed with mantle cell lymphoma [19]. 

We are uncertain of the role that chemotherapy played in this patient's presentation. While prior reports have described appendicitis in leukemic and other patients with malignant neoplasms undergoing chemotherapy, these cases are usually in association with neutropenia. This case is unusual, in that while the patient was undergoing therapy he was also receiving growth factor therapy (pegfilgrastim) and was not neutropenic. At the time of the surgery the degree of appendiceal involvement by lymphoma was not extensive; however, the possibility that the tissue had been more extensively involved prior to chemotherapy and that some component of cytoreduction contributed to the perforation of the appendix cannot be excluded. The majority of the gross and histologic features were otherwise typical of a ruptured suppurative appendicitis, however. It is also noted that acute appendicitis has been previously associated with benign lymphoid hyperplasia that may occlude the lumen of the appendix and, therefore, contribute to the development of the infection [1]. However, given the relatively limited involvement of the appendiceal submucosa by lymphoma in this case, we cannot completely rule out that the mantle cell lymphoma present in the appendix represents a true incidental finding, unrelated to the development of the acute appendicitis.

In summary, we describe an unusual case of partially treated mantle cell lymphoma presenting as an acute appendicitis. In addition to supporting the practice of routine histologic evaluation of all appendectomy specimens, this case emphasizes the potential clinical complications of chemotherapeutically treating a patient whose gastrointestinal system is involved by lymphoma. Given the frequency of involvement of the gastrointestinal tract in mantle cell lymphoma patients, this case emphasizes the importance of incorporating Cyclin D1 immunostaining into the evaluation of even morphologically unremarkable aggregates of lymphocytes in tissue samples from these patients. Furthermore, we suggest that immunohistochemical evaluation (including cyclin D1) may be indicated in all adult patients with appendicitis whose lymphoid tissue appears unusually prominent or exhibits atypical histologic features.
